# Pathergy Phenomenon

**DOI:** 10.3389/fmed.2021.639404

**Published:** 2021-05-25

**Authors:** Tulin Ergun

**Affiliations:** Department of Dermatology, Marmara University School of Medicine, Istanbul, Turkey

**Keywords:** intraepidermal pustule, Behçet's disease, Th1 response, skin pathergy reaction, cutaneous innate immune response

## Abstract

Skin pathergy reaction (SPR) is a hyperreactivity response to needle induced trauma which is characterized by a papule or pustule formation, 24–48 h after sterile-needle prick. It is affected by a wide array of factors, including the physical properties of the needles being used, number of pricks and disease related factors such as male gender, active disease. There is a great variation in reactivity among different populations with very low positivity rate in regions of low prevalence like Northern Europe, and higher prevalance mainly in communities living along the historical Silk Road, like Turkey, China and Middle Eastern countries. SPR is not constant during the disease course, has lost its sensitivity over decades and can be positive in various disorders including Sweet's syndrome, pyoderma gangrenosum, Crohn's diesease, A20 haploinsufficiency, deficiency of IL-1-receptor antagonist and few others. Nevertheless, it is a criteria included into many currently used diagnostic or classification criteria for Behçet's disease. Although, not being fully uncovered yet, available data points to the activation of both innate and adaptive immune system with an inflammatory response mediated by polymorphonuclears and T-cells. In addition to its utility in diagnosis of Behçet's Disease, SPR may serve as a model for investigating the inflammatory pathways involved in the etiopathogenesis of this complex disease.

## Introduction

Behçet's disease (BD) is a relapsing inflammatory disease with mainly mucocutaneous, ocular, vascular, gastrointestinal and neurological manifestations. Skin pathergy reaction (SPR) is a non-specific hyperreactivity response to sterile-needle-induced tissue damage. Although such response can be seen in several other diseases, it is a characteristic feature of Behçet's disease (1, 2). A positive skin pathergy reaction is defined as an erythematous papule or pustule at the site of the needle prick, resolving in 3–4 days (3). SPR is an intriguing reaction with several important aspects. It is used for diagnosis, indicates an active disease, and also serves as a model for research as clinical and histopathological findings resemble non-follicular papulopustular lesions of BD.

This review aims firstly, to address the technique of pathergy testing and determinants of positive reaction, next pathophysiology, with the light of histopathological and immunohistochemical findings and finally the diagnostic role of SPR in BD.

## Technique of Pathergy Testing

**Figure 1:**
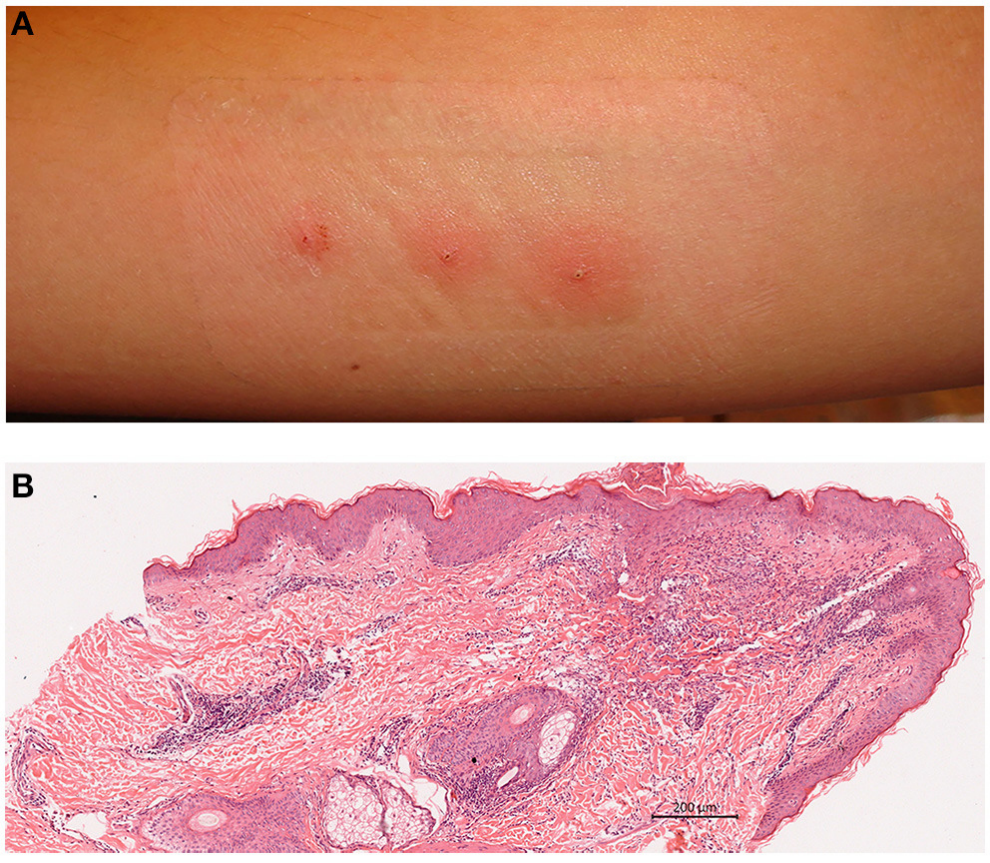
**(A)** A positive pathergy reaction evaluated at 48 h; pustules on erythemotous base. **(B)** Histopathology of positive pathergy site (haematoxylin eosin). Superficial and deep dermal perivascular inflammatory infiltrate with lymphocytes and neutrophils (Courtesy of Professor Nesimi Büyükbabani).

There is no consensus on the methodology of pathergy testing. Sharp or blunted needles, 4–6 needle pricks are used and response is evaluated after 24–48 h. Glabrous skin of both forearms are cleansed with an antiseptic, commonly alcohol, and 20 G needles are inserted either perpendicularly or oblique through the skin. Needles can be blunted by hitting against steril plastic sheath cover. Once the needle is placed into the dermis, it can be twisted couple of rounds to increase trauma. A total of 4–6 pricks are performed (Supplementary Material). Although test can be read at 24 or 48 h, 48th hour reads have been shown to increase specificity. Test is evaluated by naked eye and an erythematous papule ≥2 mm or a pustule is regarded as positive reaction (Figure 1A) (4–6).

## Determinants of Pathergy Positivity

Pathergy positivity is influenced by various factors, namely, sharpness and size of the needle, number of pricks, disease related factors, method of disinfection, medications being used and ethnic/geographic background (4, 7, 8). The impact of sharpness and size of the needle has been investigated by a few comparative studies. In one of the largest studies conducted by Dilşen et al. 20 and 26 G blunt needles were used to induce pathergy in 92 BD patients, 128 diseased and 64 healthy controls. Significantly higher responses were obtained with 20 G needles. The authors stated that, sharp needles decreased both sensitivity and intensity of reaction (9). This data was supported by the findings of Ozarmagan who have tested 92 BD patients with 20 and 26 G needles and found higher positivity rates with 20 G needles reaching to 65% (10). Regarding the sharpness, Karadag et al. have compared blunt and sharp needles and found significantly high rate of positive tests in areas where blunt needles were used (85% vs. 32.5%) (11).

Another determinant is the number of punctures. Although consensus on the number is lacking, increasing the number of pricks increases sensitivity with 19, 28, and 33% when 2, 4, and 6 pricks are performed, respectively (7). Thus, 2–3 punctures to each forearm is commonly used by physicians (6). Taken together, these consistent results on both needle sharpness and repetition, provide valid data on the impact of technique on the outcome of SPT.

## Disease Related Factors

The relation between disease characteristics and SPR has been studied by various authors. Yazici et al. reported SPR to be irrelevant from disease activity (12). On the contrary, Gilhar et al. repeated the test in active and inactive stages of the disease, and found that 90.9% of those with active disease had positive results (13). In line with their findings, two independent studies revealed significantly higher positivity rates in patients with active disease (11, 14). Since pathergy reaction is an exaggerated inflammatory response, not being constant over the disease course, a relation with disease activity may be expected. Apart from disease activity, higher reactivity has been reported among males and patients with oral aphthosis, pseudofolliculitis and uveitis (15, 16). More severe disease course and higher frequency of ocular involvement in males, may account for this relation.

The method of skin cleansing, which differs among physicians may also influence SPR. Fresco et al. have shown surgical cleansing with povidone iodine or 100% chlorhexidine solution for 4 min before testing to decrease positive yields significantly. Interestingly 4% chlorhexidine, which is commonly used for skin antisepsis failed to have an impact on results (17). Authors concluded microorganisms or chemical components of skin like sebum may account for SPR. Hence, Ozden et al. investigated different methods of pathergy testing in clinical practice and found 23.5% of physicians performed the test without prior disinfection of the skin to maximize positive test (6).

## Improving Sensitivity of SPT

Since SPR is the only available diagnostic test for Behcet's disease various attempts have been made to improve its sensitivity. Gilhar et al. have compared saline and histamine injections and found both equally provoking the reaction and leading to similar histopathologic features (13). In another study by Dilşen et al. saline injection or intravenous insertion failed to be superior to intradermal prick (9) The reactivity of oral pathergy test, being performed by insertion of a 20 G blunt disposable needle to lower lip mucosa, was also less sensitive than intradermal test (18). In a recent study Yildizhan et al. compared the sensitivity of intradermal punctures with a three-step pathergy test. In addition to intravenous puncture to the antecubital vein, saline was injected intramuscularly to glutea on three consecutive days and the puncture sites were evaluated at 24 and 48 h. Although sensitivity of three step pathergy was higher than intradermal test, with rates of 43.3 and 30%, respectively, the necessity to visit the hospital for 5 days was a major drawback (19).

Other than histamine and saline, microbial or chemical compounds were used to induce SPR. Injection of monosodium urate (MSU) crystals revealed a greater sensitivity compared to the classical pathergy test. However, unlike classical pathergy response with papular or pustular lesions, the reaction to MSU was characterized by erythema (20). More recently, Deniz et al. induced pathergy through using 20 G needle or 21 G lancet in a group of active and inactive BD patients as well as controls. The investigators inserted the needle/lancet to one site and also injected 23 valent polysaccharide pneumococcal vaccine to another site with either 20 or 21 G needles. Tests were read at both 24 and 48 h. Accordingly, injection of pneumococcal vaccine by 20 G needles revealed highest sensitivity and specificity, 64.3 and 100% respectively. Sensitivity was even higher among patients with active disease (80.3%) and specificity remained high (100%). Immunosuppressive use had a negative effect on responsiveness (21). Taken together, these data shed light on variables influencing SPT and also provide encouraging information supporting the possibility to improve sensitivity by using microbial antigens.

## Ethnic Variation

Regarding the geographic/ethnic background, there is a great variation in positivity across studies from different countries with very low positivity rates in regions of low prevalence. Neither the technique (2–6 punctures) or patient related factors (disease activity and treatment) are uniform across studies and positive reaction is reported to range between 7.7 and 84%. In a historical study from Turkey, a country with one of the highest prevalances, Tuzun et al. reported 84% of BD patients having a positive reaction (22). Reactivity rates reported in other high-prevalence countries are 62% in China, 44% in Japan, 71% in Iraq, 62% in Iran, 62% in Egypt, and 77% in Morocco. In contrast, studies from low prevalence countries such as Denmark and Sweden, revealed positive reactions in 7.7 and 8.3% of BD patients, respectively (23, 24). Other studies revealed positive SPR in approximately 15% of Korean, 20% of Jordan, 30% of British, 31% of German, and up to 60% of Turkish patients with Behçet's disease (25, 26). Although methods of studies are not uniform, one possible explanation of this variation maybe the heterogeneity of disease phenotypes in different populations.

In addition to ethnic variation, positivity rate was shown to decline over the last few decades. Davatchi et al. reported positive SPT in 61.5 and 41% of patients having disease onset before 1977 and after 1998, respectively (26). This change, lowering the sensitivity and diagnostic value of SPR, can be attributable to the use of disposable needles in post-AIDS era which are less traumatic than the non-disposable ones. Considering the relation between disease activity and SPT, another explanation maybe a trend toward milder disease over time which was shown by various studies from different populations (27–29).

## Histopatological and Immunohistochemical Findings

Main histopatological findings are mixed dermal inflammatory cell infiltration with lymphocytes, neutrophils and sparse eosinophils, condensed at perivascular sites (Figure 1B). In a controlled chronologic study we have found mainly polymorphonuclear perivascular infiltrate and intraepidermal pustules being evident as early as 4 h following prick, gradually increasing in density with a peak at 24 h with adjunctive mononuclear infiltrates. Additional patients developed intraepidermal pustules at 24 h. The inflammatory infiltrate remained constant or eventually decreased by 48 h. No change in mast cell numbers or findings consistent with vasculitis were detected. Patients with recurrent aphtous stomatitis and healthy controls also had a mixed inflammatory cell infiltrate which was sparse, however, intraepidermal pustule formation was not evident in these groups (30). Consistently, Haim et al. reported superficial perivascular mixed infiltrate with clusters of neutrophils, lack of true vasculitis and immune-complex deposition (31). Likewise, Gül et al. obtaining biopsies at 48 h, also demonstrated superficial perivascular mononuclear cell infiltrate, consisting of mainly CD3(+) T lymphocyes, intraepidermal pustules, normal mast cell numbers and abscence of vasculitis (32).

In addition to abovementioned features, several authors reported vasculopathy and vasculitis. A histopathological and electron microscopic analysis by Bang et al. revealed superficial perivascular inflammatory cell infiltrate with co-existent vascular obliteration and endothelial proliferation (33). Nazzaro et al. reported endothelial swelling and thickening of small dermal vessels (3). In their study of 11 patients, Gilhar et al. found perivascular mononucleer infiltrates with small clusters of neutrophils, sparse eosinohils and increased number of mast cells. Only two of the biopsies had the features of leucocytoclastic vasculitis whereas none had either immunoglobulin or complement deposition (13). Leucocytoclastic vasculitis or Sweet's syndrome-like changes were found 24 h after histamine injections by Jorizzo et al. (34). Taken together, histopathological spectrum points to a mixed inflammatory infiltrate and intraepidermal pustules one end, findings of vasculopathy and true leucocytoclastic vasculitis on the other end. This disparity maybe due to disease activity, organ involvements, medication use, taking biopsy at different timepoints, effects of histamine, and ethnic background.

Immunohistochemical examination of pathergy site at 48th hour revealed HLA-DR expression of keratinocytes and inflammatory cells, ICAM and e-selectin expression by endothelial cells. Inflammatory infiltrate had a dominance of CD3(+), CD4(+), CD45Ro(+) cells and small collections of neutrophil elastase positive cells were detected in needle insertion sites (32). A mixed perivascular inflammatory cell infiltrate extending into the deep dermis without vasculitis and endothelial E-selectin, P-selectin and CD 105 expressions were additional findings (35). Aiming to investigate inflammatory mediators at skin lesions including pathergy sites, Ben Ahmed et al. showed significant increases in the messenger RNA expression of interleukin-8, monocyte chemoattractant protein 1, interferon-γ, IL-12 and IL-10 in BD lesions compared with normal skin. The authors concluded a strong Th1 polarization with IL-10 probably having a role in preventing a more severe inflammatory response (36). In a comprehensive study, Melikoglu et al. investigated cellular and molecular elements of inflammatory response to needle prick in BD and healthy control subjects at 0, 8, and 48 h. Unlike controls, BD patients had increased influxes of mature dendritic cells, monocytes, lymphocytes including T regulatory cells by 48 h. Similarly, increases in cytokines IFN-γ, IL-12 p40, IL-15, IL-10, IFN-γ induced genes and transcription factors were found. Chemokines, MIP3-α, IP-10, Mig, iTac leading to recruitment of dendritic cells, mononuclear cells as well as adhesion molecules (ICAM-1, VCAM-1) were noted in SPR site of BD patients but not in the skin of normal controls. These results also support an exaggerated lymphoid Th1-type response in SPR (37). Alpsoy et al. investigated androgen receptor index of SPR site as compared to non-lesional skin and found higher expression among males, concluding androgens having a possible role in reaction (38).

## Pathergy Reaction in Organs Other Than Skin

The pathergy reaction is not limited to skin in BD patients and a similar hyperreactivity response can be observed following mechanical or surgical trauma in various organs and tissues. BD patients may have exacerbation of uveitis following eye surgery or intraocular injections as well as synovitis following arthrocentesis. Angiographic interventions or vascular surgery may lead to arterial thrombus or aneurysm, venipuncture may induce superficial thrombophlebitis, segmental bowel resection may trigger intestinal ulcers (39–42). Other examples of pathergy phenomenon are, appearance of oral ulcers following dental therapuetic interventions and placement of othodontic braces and also pustular lesions after laser hair removal (43, 44). These findings support the notion that hyperinflammatory response triggered by trauma is a feature of the disease itself, rather than being an organ-specific phenomenon and investigating SPT sites, may help in understanding inflammatory pathways involved in etiopathogenesis of BD.

## Diagnostic Value

Pathergy reaction can be seen in pyoderma gangrenosum, Sweet's syndrome, deficiency of IL-1-receptor antagonist (DIRA) and Crohn's disease (5, 45–47). There are also case reports of pathergy in atypical eosinophilic pustular folliculitis, neonates with Down's syndrome, myeloproliferative disorders, non-Hodgkin's lymphoma and chronic myeloid leukemia treated with interferon-α (5, 48). A20 haploinsufficiency (HA20), a disease with BD-like mucocutaneous, articular, gastrointestinal, and ocular symptoms is a new addition to the list of disorders with positive SPT (49). Although the prevalance of these disorders, as well as positive pathergy yield is low, they hamper specificity. Other downsides are, lack of standardization in performance and evaluation, low reproducibility, remarkable decrease in sensitivity over decades and ethnic variation in positivity rates, altogether limiting the usefulness of this test in the clinical setting.

The role of SPR in diagnosis of BD varies according to the diagnostic criteria set being used. In International Study Group classification criteria, SPR is one of the five components. SPR is also included in International Criteria for Behçet's Disease at which ≥4 points are required to fulfill the criteria (50). Conversely, SPR is excluded in Behcet's Syndrome Japanese Criteria, as positivity is low among Japanese BD patients (51). Recently, an international expert group analyzing clinical manifestations of 219 pediatric BD patients, proposed consensus classification criteria for pediatric Behçet's disease (PEDBD), which excluded SPR. These proposed criteria had a sensitivity and specificity of 91.7 and 42.9%, respectively and the addition of the SPR failed to improve the performance (52). The impact of the positive pathergy test on the performance of 16 available classification/diagnosis criteria sets for Behcet's disease was analyzed. Accordingly, without SPR, 15 out of 16 criteria set lost sensitivity, gained specificity, and lost accuracy, highlighting the diagnostic value of this test (53).

In spite of abovementioned drawbacks, pathergy test is an easy and cheap test to perform and its positivity beyond aiding diagnosis also indicates an active disease and hence is used widely in many clinics.

## What Induces SPR?

The mechanisms underlying the augmented immune response in positive SPR sites are not understood yet. Is SPR a non-specific hyperinflammatory response to traumatic insult? Is it an altered/exaggerated response to microbial or epidermal antigens? Or is it an dysregulated wound healing response?

Higher response rate with blunt and/or large needles favors the possibility of reaction to mechanically damaged epidermal and dermal components. This is further supported by the fact that sterile areas such as joints, eye, blood vessels also develop hyperinflammatory response with trauma. Human skin, well equipped with cellular and molecular components of innate immune system, is capable of immediately responding to danger signals induced by microbial invasion and tissue damage. Koebner phenomenon, which is the appearance of specific lesions in uninvolved skin as a consequence of trauma, is a well-known feature of various skin disorders, mostly psoriasis, vitiligo and lichen planus. Injured epidermal and dermal cells produce various chemokines, cytokines, growth factors, antimicrobial peptides altogether leading to an inflammatory response (54, 55). In fact, Sjögren et al. have shown minimal trauma through insertion of sterile microcatheters to healthy individuals' uninvolved skin to induce proinflammatory cytokines, IL-1b, IL-6 and IL-8. These cytokines have reached peak skin levels at 3–8 h, declining thereafter, albeit existing in small amounts, at 24 h (56). This sequence overlaps with findings of histopathological studies at which inflammatory infiltrate was evident by 4 h, reaching a peak at 24 h and then declining (30). These data underlines the role of trauma induced skin damage to activate cutaneous innate immune response, which is somewhat exaggerated due to genetic and environmental factors in BD patients.

The hypothesis on the role of microbial antigens as triggers is supported by decline in response when skin is surgically cleansed by 100% chlorhexidine or povidone iodide. Also exacerbation of oral aphthae, seen following dental invasive treatments can be due to oral microorganisms (44). Increased response to self saliva containing various microorganisms and significantly higher sensitivity with pneumococcal vaccine also support the notion of microbial elements as the inducers of SPR (21). Nonetheless, no specific bacterial antigen has been shown until now. Finally, an altered wound healing is claimed to cause SPR. Nevertheless, wound healing process in patients with BD has been shown to be unaltered (57).

Aiming to generate a hypothesis on the cascade of events ending with inflammation in pathergy sites, one can claim SPT to be initiated by trauma induced keratinocyte damage and/or insertion of yet undefined microbial antigen into the skin. Expression of Toll-like and NOD-like receptors capable of binding danger and pathogen associated molecular patterns by keratinocytes cause activation of intracellular signaling pathways, release of IL-6, TNF-α and IL-1β. These cytokines activate dermal dendritic cells, which in turn lead to Th1 responses through releasing IL-12, IL-23 and INFs (58). Keratinocytes also release chemokines CXCL8-11 which attract neutrophils, mature dendritic cells and activated T-lymphocytes to the dermis eventually causing collections of polymorphonuclear cells, dermal mixed inflammatory infiltrate as seen in histopatological examination (37).

## Future Directions

Much remains unknown about the immunopathogenesis of BD and SPR may serve as an *in-vivo* model for investigating sequentially, molecular and cellular elements of immune response. Using novel non/minimally invasive research techniques such as skin dialysis or microneedle pathes for sampling cellular and molecular mediators of inflammation may cast light on inflammatory pathways. Future research should focus on methods of improving sensitivity of SPR, identifying possible triggers, effector dendritic cell subtypes, T cell repertoire and also mechanisms of dysregulated intrinsic tolerogenic mechanisms. In the era of biologics and small molecules, such work may enable development of targeted therapies.

## Author Contributions

TE: concept and design, literature search, and writing manuscript.

## Conflict of Interest

The author declares that the research was conducted in the absence of any commercial or financial relationships that could be construed as a potential conflict of interest.

## Supplementary Material

Supplementary Video 1 Pathergy technique. Cleansing forearm skin with chlorhexidine 4%, insertion of 20 G needles oblique through the skin, with 3 pricks for each forearm, twisting needles a couple of rounds to increase trauma and covering the area with gauze.
